# Prevalence of musculoskeletal symptoms in hospital nurse technicians and licensed practical nurses: associations with demographic factors

**DOI:** 10.1590/bjpt-rbf.2014.0026

**Published:** 2014

**Authors:** Roberta F. C. Moreira, Tatiana O. Sato, Fabiana A. Foltran, Luciana C. C. B. Silva, Helenice J. C. G. Coury

**Affiliations:** Departamento de Fisioterapia, Universidade Federal de São Carlos (UFSCar), São Carlos, SP, Brasil

**Keywords:** occupational health, epidemiology, exercise, physical therapy

## Abstract

**Objective:**

: This cross-sectional study aimed at analyzing: 1. the main musculoskeletal symptoms (MSS) presented by hospital nursing workers and; 2. personal, occupational, and health factors related to MSS among them.

**Method:**

: Two questionnaires were filled in by 245 nurse technicians (NTs) and licensed practical nurses (LPNs) (response rate 95%) associated with direct patient care sectors from a hospital. These questionnaires were: the standardized version of the Nordic Musculoskeletal Questionnaire (NMQ) and one including questions on 15 demographic independent variables potentially related to outcomes from the NMQ. Univariate analyses and binary logistic regression analyses were performed to identify which variables would explain the occurrence of MSS in different body regions.

**Results:**

The low back (57%), shoulder (52%), and neck (48%) were identified as the most affected regions. The logistic regression analysis showed that low back symptoms in the last 12 months were significantly associated with LPN activities (OR=2.36; CI=1.24-4.5) and previous sick leave due to MSS (OR=5.97; CI=1.2-29.1). Smoking was significantly associated with symptoms in the low back (OR=2.77; CI=1.13-6.8) and thoracic spine (OR=2.37; CI=1.04-5.40). Physical exercise showed a protective effect on the cervical spine (OR=0.42; CI=0.23-0.77). Previous sick leave was significantly associated with pain in the knees (OR=4.24; CI=1.33-13.5) and in the upper limbs (OR=5.36; CI=1.07-26.7).

**Conclusions:**

The nursing workers who were evaluated presented a high prevalence of MSS. Previous history of sick leave was strongly associated with the presence of symptoms in various body regions. These results indicate the need for preventive programs in the hospital environment in order to control more severe MSS in nursing professionals.

## Introduction

Work-related musculoskeletal disorders (WRMDs) are responsible for early exit from the labor market^1^ ^,^ ^2^ and represent the most common cause of absenteeism among workers^3^ ^,^ ^4^. In this context, physical therapy plays an important role as an intervention which can reduce the need for more costly or invasive procedures, thus preventing diseases and promoting health^5^.

WRMDs are highly prevalent among nursing professionals^6^ ^-^ ^8^ and the most frequent complaints are low back pain, with a prevalence rate of 30 to 60%^6^ ^,^ ^7^ ^,^ ^9^ ^-^ ^13^, followed by the neck and shoulder symptoms, with prevalence rates of 30 to 48% and 43 to 53%, respectively^9^ ^,^ ^11^ ^-^ ^14^.

Various epidemiological studies have reported an association between work overload and musculoskeletal disorders^6^ ^,^ ^10^ ^,^ ^15^ ^-^ ^17^. In addition to ergonomic factors, psychosocial risk factors such as high demand, low job control, and lack of social support have also been recognized as contributing factors to the development of musculoskeletal disorders among nursing professionals^9^ ^,^ ^10^ ^,^ ^16^ ^,^ ^18^. This multifactorial nature of the disorders shows the need for risk factor evaluations that consider a high number of potential contributing factors simultaneously^9^ ^,^ ^19^. However, due to the multifactorial origin of these disorders^20^ ^,^ ^21^, the relationship between demographic characteristics (gender, age, height, weight, job, work sector, time in current sector, smoking, physical exercise, etc.) and the presence of musculoskeletal disorders has not yet been clarified^7^ ^,^ ^9^ ^,^ ^16^.

Considering the importance of broadening epidemiological knowledge related to MSS among nursing professionals^22^ and the need to evaluate these symptoms in a broader context for future preventive and therapeutic programs, the objectives of this study were to investigate: 1) the main symptoms presented by nurse technicians (NTs) and licensed practical nurses (LPNs) and 2) the simultaneous relationship between personal, occupational, and health factors possibly related to the presence of symptoms in different body regions.

## Method

The present epidemiological study followed the STROBE methodology^23^ of conducting observational epidemiology studies.

### Study design

A cross-sectional epidemiological study was carried out to evaluate the prevalence of MSS among NTs and LPNs from a Brazilian hospital and to identify the potentially related factors.

### Location of the study

The study was carried out in a hospital in the state of São Paulo, Brazil. The questionnaires were distributed during the work shifts in sectors involving direct patient care. The participants incurred no expense and received no compensation.

### Participants and inclusion criteria

Brazilian nursing teams are basically comprised of three occupational groups: nurse technicians, licensed practical nurses, and registered nurses. In Brazil, NTs and LPNs represent most of the nursing workforce. These workers are mainly responsible for activities that involve direct contact with patients and, for this reason, are quite exposed to physical risk factors. Thus, the present study evaluated NTs and LPNs only.

Federal Law 7498/86^24^ regulates the activities performed by these professionals and states that NTs and LPNs are responsible for most of the direct care of patients. However, activities carried out by NTs require a lower level of decision-making than the ones performed by LPNs and involve mid-level tasks of a repetitive nature.

All of the NTs and LPNs associated with direct patient care at the hospital were invited to participate in the study (n=292); they worked regularly in either day shifts (7:00 am to 7:00 pm) or night shifts (7:00 pm to 7:00 am). The adopted inclusion criteria were: to be registered as an NT or LPN; work in the department responsible for direct patient care, and to be employed for at least 12 months. All participants signed the informed consent form and the research procedures were approved by the Human Research Ethics Committee of Universidade Federal de São Carlos (UFSCar), São Carlos, SP, Brazil (CAAE: 1080.0.00.135-10).

### Hospital department characteristics

A hospital's emergency department is accessible to the general population and is designed to assist patients with or without risk of death who require immediate health care^25^. Patient referral is carried out according to the complexity of the cases treated. Simple cases are dealt with at the emergency care units and more complex cases are sent to other units of the hospital. Hospital wards are departments for patients who do not need constant observation. One companion is allowed to stay with each patient all the time. Intensive Care Units (ICUs) are departments in which high level technology equipment is used for the care of critically ill patients. ICU patients need constant observation, as well as continuous medical and nursing care^25^. In these departments the circulation of both staff and visitors is restricted and controlled. It is important to emphasize that the physical and mental demands of each department vary due to the different levels of assistance, complexity, technology, and nurse-patient relationship of each department.

### Evaluated variables and data sources

Two questionnaires were applied: the standardized Nordic Musculoskeletal Questionnaire (NMQ) and a questionnaire specifically designed for the present study that included 15 independent variables potentially related to the response variables of the NMQ^26^. In the customized questionnaire, personal, occupational, and health factors were included based on relevant, previously published epidemiological studies about risk factors^10^ ^,^ ^27^ ^,^ ^28^ and on the authors' own experience^29^ ^-^ ^31^. The questions were structured as direct queries. Pilot tests were run before the questionnaires were applied to evaluate the clarity of the content and time taken to respond to the questions.

The following information was covered by the questionnaire: 1) occupational aspects - work department (emergency room, hospital wards or intensive care), shift (day or night), job position (NT or LPN), time in this position (years), time at the institution (years), other paid activity (yes or no); 2) personal characteristics - gender (male or female), age (in years), body mass index classification (normal, overweight, obese), marital status (married or single), children (yes or no), routine housework (yes or no); 3) health condition: regular physical activity (yes or no), smoking (yes or no), and history of sick leave of more than 15 days due to musculoskeletal disorders (yes or no).

The Brazilian version of the NMQ^32^ was used to identify the presence of symptoms in the previous 7 days and previous 12 months in different regions of the body, the impairment these symptoms caused in daily life activities (DLAs) and whether or not medical assistance was sought for the symptom. The questionnaires were answered by the workers during their work shift. It should be mentioned that there was no interference from superiors or compensation for the workers.

### Independent and dependent variables

The discrete independent variables: age, time in the current job position, and time at the institution were categorized according to quartiles (Table 1). The BMI values were categorized as: 1) normal (≤25), 2) overweight (>25 and <30), and 3) obese (>30)^33^.

**Table 1:** Categorization of quantitative variables according to quartiles.

Categories	Age (years)	Time employed in the current position (years)	Time employed at the institution (years)
Category 1(≤25^th^)	≤26	≤2	≤1.5
Category 2 (>25^th^ and ≤50^th^)	>26≤34	>2≤5	>1.5≤4
Category 3 (>50^th ^and ≤75^th^)	>34≤42.5	>5≤14	>4≤10
Category 4 (>75^th^)	>42.5	>14	>10

All dependent variables were dichotomous (presence or absence). Variables related to neck, thoracic spine, and lumbar spine symptoms were grouped under the term "spine segment". Variables related to shoulder, elbow, wrist, and hand symptoms were grouped as "upper limb (UL) segment". Variables related to hip, thigh, knee, ankle, and foot symptoms were grouped as "lower limb segment". The dependent variable "symptoms in any body region" corresponded to the nine body regions evaluated by the NMQ.

### Controlling sources of bias

Initial clarification was given to all participants to prevent misunderstandings in their responses. If asked, additional information was provided individually, avoiding interpretations or any other form of inducement toward particular responses.

### Sample size

All NTs and LPNs who were present (i.e. not on leave, vacation or day off) when the evaluation took place (n=292) were evaluated. The final sample consisted of 245 workers who matched the study's inclusion criteria.

### Statistical methods

The data were descriptively analyzed by calculating the frequencies, quartiles, means, and standard deviation. A univariate analysis was carried out with the chi-square association test (χ^2)^. The independent variables significantly associated (*P*≤0.25) with the dependent variables were included in a logistic regression model^34^. The objective of the logistic regression analysis was to identify which variables explain the occurrence of musculoskeletal symptoms in different body regions. The data were analyzed in SPSS 11.5.

## Results

### Subjects

Out of the 292 LPN and NT active workers in the direct patient care sectors, 258 matched the study inclusion criteria. Thirteen workers did not participate because on the day of data collection they either had the day off (n=8), were absent from work (n=4) or they were unavailable to answer the questionnaire (n=1). Therefore, the sample included 245 individuals, representing 95% of the eligible subjects. There were 226 women and 19 men; the mean age was 35.5 years old (±10.7; min. 19 and max. 68). The mean time that the participants had been employed in their current position was 8.6 years (±8.5; min. 1 and max. 47) and the mean time at the institution was 6.8 years (±7.3; min. 1 and max. 47). The demographic characteristics of the sample (n=245) regarding occupational, personal, and health aspects are presented in Table 2.

**Table 2:** Demographic characteristics of the sample regarding occupational, personal, and health aspects.

Occupational aspects		*N* **(%)
Job	Nurse technician	168 (68.6%)
	Licensed practical nurse	77 (31.4%)
Work shift	Day	134 (54.9%)
	Night	111 (45.1%)
Job sector	Emergency room	21 (8.6%)
	Hospital wards	161 (65.7%)
	ICUs	63 (25.7%)
Time in this position	up to 2	79 (32.2%)
(years)	+2 to 5	50 (20.4%)
	+5 to 14	60 (24.5%)
	+14	56 (22.9%)
Time at the institution (years)	Up to 1.5	65 (26.5%)
	+1.5 to 4	65 (26.5%)
	+4 to 10	60 (24.5%)
	+10	55 (22.5%)
Other paid activity	Yes	61 (25.2%)
	No	184 (74.8%)
**Personal aspects**		***N*** ** **(%)**
Gender	Female	226 (92.2%)
	Male	19 (7.8%)
Age (years)	Up to 26	62 (25.3%)
	+26 to 34	58 (23.7%)
	+34 to 42.5	56 (22.8%)
	+42.5	59 (24.2%)
Body mass index	Normal	91 (44.8%)
	Overweight	63 (31%)
	Obese	49 (24.2%)
Marital status	Single	122 (50%)
	Married	122 (50%)
Children	Without	93 (38.4%)
	With	149 (61.6%)
Housework	Performs	224 (91.8%)
	Does not perform	20 (8.2%)
**Health aspects**		***N*** ** **(%)**
Regular physical exercise	Yes	72 (29.4%)
	No	173 (70.6%)
Smoker	Yes	34 (13.9%)
	No	210 (86.1%)
Previous sick leave due to musculoskeletal symptoms	Yes	14 (5.8%)
	No	226 (94.2%)

The evaluated population consisted predominantly of women (92.2%) who did not exercise regularly in their free time (70.6%) and were exposed to double work shifts due to housework (91.8%). Most subjects (55.2%) were in the overweight or obese categories and approximately 53% had been nurses for less than 5 years.

The number and percentage of symptomatic workers evaluated by the NMQ, as well as for the categories: 'spine', 'UL', 'lower limb', and 'at least one body region' are presented in Table 3.

**Table 3:** Proportion of symptomatic subjects for the body regions evaluated by NMQ (n=245).

Body region	Symptoms in the last 12 months (%)	Impairment in DLAs (%)	Seeing a physician due to symptoms (%)	Symptoms in the last 7 days (%)
At least one region	229 (93.5)	68 (27.8)	95 (38.8)	157 (64.1)
Cervical spine	117 (47.8)	22 (9)	17 (7)	55 (22.4)
Thoracic spine	120 (50.8)	19 (7.8)	26 (10.7)	62 (25.3)
Lumbar spine	140 (57.1)	29 (11.8)	35 (14.3)	83 (33.9)
Spine	187 (76.3)	44 (18)	56 (22.9)	121 (49.4)
Shoulder	127 (52)	16 (6.5)	26 (10.7)	58 (23.8)
Elbow	19 (7.8)	3 (1.2)	6 (2.4)	6 (2.4)
Wrist and hand	78 (31.8)	10 (4.1)	16 (6.5)	32 (13.1)
Upper limb	152 (62)	23 (9.4)	40 (16.3)	76 (31)
Hip and thigh	80 (32.7)	9 (3.7)	16 (6.5)	35 (14.3)
Knee	78 (31.8)	16 (6.5)	15 (6.1)	30 (12.2)
Ankle and foot	99 (40.4)	19 (7.8)	23 (9.4)	52 (21.2)
Lower limb	160 (65.3)	31 (12.7)	43 (17.6)	85 (34.7)

Table 3 shows the high prevalence of MSS in at least one body region among the evaluated nursing professionals, both in the last 12-month and seven-day periods. The symptoms led the worker to seek medical assistance and impaired the performance of DLAs in approximately 1/3 of the individuals affected.

Analysis of the symptoms according to the body region showed that during the previous 12 months the spine was the most affected part in 3 out of 4 individuals evaluated, followed by the lower limbs and the ULs. Considering the regions individually, the lumbar spine, shoulder and cervical spine were the regions with the highest prevalence of symptoms in the previous 12 months, followed by the thoracic spine and the ankle and foot regions.

Regarding the effects of symptoms on the performance of DLAs, more than 1/4 of the individuals experienced some impairment. The lumbar region was the most critical, followed by the cervical spine, thoracic spine, ankle, and foot. Among the professionals evaluated, the spine was identified as the part that most affected the DLAs. Symptoms in at least one body region led more than 1/3 of the participants to seek medical assistance, and symptoms in the lumbar region were the most prevalent.

The logistic regression showed the variables associated with the presence of MSS in the evaluated population. The results of this analysis are presented in Table 4.

**Table 4:** Factors associated with the presence of musculoskeletal symptoms based on analysis of the binary logistic regression

Body Region	Factor	b	SE	Wald	p	OR	CI (OR)	*R* ^*2*^ **	c^2^ (df*)*
**Symptoms in the last 12 months**									
*Cervical spine*	Physical exercise	–0.862	0.930	7.848	0.005	0.422	0.231-0.772	0.010	17.63 (7)*
*Thoracic spine*	Smoking	0.863	0.420	4.213	0.04	2.369	1.04-5.398	0.084	15.13 (6)*
*Lumbar spine*	Job position	0.861	0.329	6.855	0.009	2.364	1.242-4.503	0.120	20.19 (6)*
	Smoking	1.021	0.458	4.973	0.026	2.775	1.132-6.807		
	Sick leave	1.787	0.809	4.885	0.027	5.973	1.224-29.142		
*Vertebral column*	Job position	0.924	0.394	5.487	0.019	2.519	1.163-5.457	0.150	25.09 (4)*
	Physical exercise	–0.981	0.334	8.609	0.003	0.375	0.195-0.722		
	Smoking	0.176	0.759	5.398	0.02	5.826	1.317-25.765		
*Wrist and hand*	Sick leave	1.641	0.594	7.638	0.006	5.159	1.612-16.514	0.090	15.84 (5)*
*Upper limbs*	Gender	–1.328	0.053	6.254	0.012	0.265	0.094-0.75	0.073	12.64 (3)*
	Sick leave	1.679	0.82	4.195	0.041	5.358	1.07-26.71		
*Knee*	Sick leave	1.445	0.592	5.957	0.015	4.243	1.329-13.542	0.074	11.9 (4)*
*Lower limbs*	Sick leave	1.723	0.804	4.598	0.032	5.603	1.16-27.1	0.090	14.25 (6)*
**Impairment in DLAs due to symptoms**									
*Any region*	Sick leave	2.054	0.631	10.585	0.001	7.797	2.263-28.87	0.100	17.65 (3)*
*Thoracic spine*	Sick leave	1.951	0.691	7.971	0.005	7.037	1.816-27.27	0.05	12.33 (3)*
*Lumbar spine*	Sick leave	3.858	1.239	9.701	0.002	47.38	4.18-53.69	0.300	29.30 (9)*
*Vertebral column*	Sick leave	2.43	0.633	14.72	0.000	11.360	3.283-39.307	0.160	23.13 (6)*
*Shoulder*	Sick leave	1.772	0.785	5.098	0.024	5.88	1.263-27.367	0.160	15.23 (5)*
*Upper limbs*	Housework	–1.637	0.606	7.298	0.007	0.194	0.059-0.638	0.110	12.43 (5)*
**Symptoms for which medical assistance was sought**									
*Any region*	Gender	–1.830	0.745	6.036	0.014	0.16	0.04-0.69	0.170	28.15 (6)*
	Sick leave	2.008	0.782	6.59	0.01	7.45	1.61-34.5		
*Cervical spine*	Sick leave	2.216	0.786	7.953	0.005	9.173	1.96-42.80	0.100	17.63 (7)*
*Thoracic spine*	Other paid activity	1.015	0.49	4.148	0.042	2.76	1.04-7.33	0.140	15.30 (4)*
	Sick leave	1.678	0.649	6.685	0.01	5.35	1.5-19.1		
*Lumbar spine*	Job position	1.217	0.469	6.745	0.009	3.378	1.35-8.46	0.230	28.24 (8)*
	Sick leave	1.94	0.65	8.97	0.003	6.954	1.95-24.74		
*Vertebral column*	Sick leave	2.58	0.724	12.683	0.000	13.18	3.18-54.5	0.200	27.62 (8)*
*Shoulder*	Other paid activity	1.081	0.519	4.332	0.037	2.947	1.065-8.155	0.250	28.17 (6)*
	Sick leave	2.263	0.715	10.02	0.002	9.614	2.36-39.04		
*Upper limbs*	Sick leave	1.576	0.59	7.04	0.008	4.836	1.51-15.5	0.100	12.33 (5)*

β - logistic regression coefficient

SE - standard error

Wald - logistic regression coefficient divided by the square SE

P - significance level of the Wald statistics

OR - odds ratio

CI(OR) - confidence interval of the 95% odds ratio

dg - degrees of freedom

* P<0.05.

The logistic regression analysis (Table 4) showed that spinal pain in the last 12 months, particularly in the lumbar region, was significantly associated with job position, i.e. LPNs presented with more symptoms. Despite the differences in work demand between departments, there was no relationship between job sector and musculoskeletal symptoms. Smoking was significantly associated with thoracic spine symptoms; physical exercise had a protective effect on the cervical spine. Pain in the lower limbs, particularly in the knees, was significantly associated with the presence of previous sick leave; and UL symptoms were significantly more frequent in women.

DLA impairment due to symptoms in different body regions, particularly the lumbar spine followed by the spine in general, shoulders, and thoracic spine, were significantly associated with a history of previous sick leave due to musculoskeletal problems (Table 4). DLA impairment due to UL symptoms was also significantly associated with housework.

Seeking medical assistance was associated with previous sick leave due to MSS in general, particularly in the cervical spine and ULs (Table 4). Having another paid occupation also led workers who experienced pain in the thoracic spine and shoulders to seek medical assistance. Job position as an LPN was associated with seeking medical assistance for lumbar pain.

## Discussion

The most prevalent body regions for symptoms in the previous 12 months were the lumbar spine, shoulders, and neck, followed by the thoracic spine and the ankle and foot region. Similar results were found in studies that used the NMQ to evaluate LPNs and NTs in Brazil^11^ ^-^ ^13^, as well as in studies from other countries with nursing assistants^7^ ^,^ ^9^ ^,^ ^18^ ^,^ ^19^ ^,^ ^35^ ^,^ ^36^.

A mean of 92.1% of the participants of these studies reported symptoms in at least one body region compared to 93.5% in the present study, indicating a very high and similar prevalence (Table 5). The percentages per region were also high and similar between the other studies and the present one: 65.8 and 57% for the lumbar spine, 50.3 and 52% for the shoulder, and 49.3 and 48% for the neck, respectively. Most of the studies in Table 5 also identified the lumbar spine, neck, and shoulder as the most prevalent regions for MSS among nursing professionals.

**Table 5:** Comparison of the prevalence of musculoskeletal symptoms among studies carried out with nursing assistants.

Country	NA Population	Lumbar (%)	Shoulder (%)	Neck (%)	At least one region	Study
Brazil	100%	57	52	48	93.5	Present study
Brazil	70%	73	62	67	96.3	Magnago et al.^13^
Brazil	100%	68	54	56	96	Barbosa et al.^12^
Brazil	100%	59	40	28	93	Gurgueira et al.^11^
Taiwan	100%	66	----	----	----	Feng et al.^36^
Turkey	75%	69	46	54	90	Tezel ^35^
Greece	40%	75	37	47	85	Alexopoulos et al.^20^
Japan	5%	54	43	31	----	Ando et al.^7^
Sweden	100%	64	60	53	----	Josephson et al.^18^
Sweden	40%	65	60	59	----	Lagerström et al.^9^

NA : Nursing Assistant

A high prevalence of MSS in the lumbar spine, shoulder, and neck regions was reported by nursing professionals^28^ ^,^ ^37^. The activities performed in direct patient care usually involve upper limb force, trunk flexion, and extension movements causing an impact on the musculoskeletal system, particularly for the spine and shoulder regions^17^ ^,^ ^35^ ^,^ ^38^. Along these lines, Tullar et al.^39^ recognized the role of patient transfer and lifting activities on the presence of musculoskeletal disorders among healthcare workers. The main risk factors for the development of musculoskeletal disorders among these workers are: pushing occupied beds, lateral patient transfers, repositioning patients in bed, making occupied beds, as well as lifting and carrying heavy equipment over long distances^40^.

Even though the results presented in Table 5 were from different countries and involve different cultures and availability of equipment, the MSS prevalence was high in all of them. Several aspects seem to contribute to this in different ways, such as mean worker age, time in job position, patient impairments, and technology available for facilitating patient transportation^39^ ^,^ ^41^.

The results of the logistic regression showed that previous sick leave due to musculoskeletal pain was strongly associated with seeking medical assistance due to MSS. Similar results were found among general workers evaluated by Haahr et al.^42^. Even though sick leave policies vary according to each country's legislation, in general, these benefits are given only after medical confirmation of the seriousness of the injury and degree of functional impairment^43^. Therefore, an association between sick leave, severe symptoms, the search for medical assistance, and DLA impairment is not surprising. Another aggravating factor is poor recovery after musculoskeletal injury. According to Rosenman et al.^44^, this is often due to the workers' lack of access to qualified rehabilitation services.

Job position was a major factor for spine-related outcomes; LPNs had a greater chance of presenting symptoms and seeking medical assistance than NTs. This subject still seems to be controversial in the literature. In a number of countries, the education level of nursing assistants is lower than registered nurses and they are acknowledged to have a greater predisposition to low back pain than registered nurses^18^ ^,^ ^35^ ^,^ ^38^. Considering that the names used to classify nursing professionals vary from country to country according to the work organization and the workers' educational level, direct comparisons between groups should be avoided. Despite this, as previously described, both NTs and LPNs perform highly demanding physical tasks. Nevertheless, LPNs are exposed to a higher cognitive overload due to accumulated activities and the greater complexity of their tasks, which could explain the present results for these two job positions.

Housework was associated with symptoms. However, this result must be interpreted with caution, since the negative value found for the β coefficient could suggest that performing housework would reduce the probability of DLA impairment by 0.194 due to UL symptoms. In fact, this association might be interpreted as an antalgic, rather than a protective factor.

Women had a 30% greater chance of developing UL symptoms than men. A review study^45^ reinforces this finding, demonstrating that women have a greater tendency to present upper MSS than men. Among several other factors, an association between housework, gender, and UL symptoms is recurrent in several studies. Nordander et al.^46^ hypothesize that the dedication of free time to housework reduces the recovery period required by the muscle groups involved at work and increases the risk of injury, particularly for physically demanding jobs, as is the case of the evaluated workers.

Regarding personal risks, smoking was identified as an important factor for symptoms in the thoracic region, lumbar region, and spine in general. Power et al.^47^ and Bejia et al.^48^ also found a positive association between lumbar pain and smoking for individuals who performed physically demanding activities. Nevertheless, Lagerström et al.^9^ found no such association in a study conducted with NTs.

It has been acknowledged that nicotine causes vasoconstriction which reduces the amount of oxygen and nutrients available to muscles, ligaments, and intervertebral discs, increasing chances for degenerative processes in the intervertebral discs^49^ and injuries^50^. Furthermore, continued smoking affects lung clearance, causing an accumulation of secretion and increasing coughing reflexes^51^, which overloads intercostal muscles and increases intra-abdominal pressure. The main biological mechanisms triggered by smoking that could explain spinal symptoms are linked to: 1) coughing reflexes; 2) increased fibrin deposition which leads to chronic inflammation; and 3) reduced blood flow and oxygenation of the tissues, which affect the metabolic balance of the discs and accelerate degenerative processes leaving the spine more susceptible to mechanical deformations and injuries^52^.

It is important to consider that, even though several epidemiological studies have reported an association between smoking and lumbar pain, factors such as the variety of definitions of lumbar pain, the multiple causes of the symptoms, and the variations in evaluation approaches and results make it difficult to come to a conclusive understanding of the literature^8^ ^,^ ^53^ and limit comparison of the results.

Among the personal aspects investigated here, some attenuating factors were identified, such as the protective effect of physical exercise against neck symptoms. This subject still seems to be controversial in the literature. Lagerström et al.^9^ identified that a poor physical condition increases the chance of cervical symptoms by 1.43, which supports the possibility that exercise has a protective effect against neck symptoms. However, other studies have reported that the incidence of neck pain in workers who exercise regularly in their free time is similar to that of those who do not^54^ ^,^ ^55^. This controversy may be related to the definition of physical exercise because when the control of this variable (exercise) is increased, its protective effect becomes more consistent.

Systematic reviews about the effects of exercise on musculoskeletal pain in active workers^29^ ^,^ ^31^ found a protective effect in the occupational environment against lumbar and cervical pain in workers with heavy and sedentary activities, respectively. Martins and Marzialle^56^ also identified benefits of therapeutic exercises for nursing workers with shoulder pain.

Additionally, a cohort study^57^ with 1,742 symptomatic and asymptomatic workers demonstrated that regular involvement in sports for at least 10 months per year reduced the risk of symptoms in the neck and shoulder regions (OR:0.82). Thus, in the case of exercise carried out regularly in an occupational environment, as well as the regular practice of sports, there was a protective effect of physical activity on musculoskeletal pain in active workers.

### Limitations and final considerations

The cross-sectional design of the present study does not allow for causal relations to be established between the symptoms and exposure to the tasks performed by NTs and LPNs. According to Punnett and Wegman^58^, another limitation associated with cross-sectional studies carried out in work environments is the selection bias due to the exclusive evaluation of active workers, which can underestimate the symptoms of the full staff as it does not include data from individuals on leave.

A positive aspect of this study was the evaluation of personal factors and their participation in work-related disorders, which has not been clearly established so far. Considering the high prevalence of MSS among the evaluated professionals and the impairments that these symptoms might cause, public policy should encourage their prevention to reduce sick leave. Stimulating physical exercise, organizing anti-smoking campaigns, controlling risk factors through ergonomic intervention, ensuring proper training and breaks are some of the measures that should also be undertaken.

## Conclusion

The LPNs and NTs evaluated in this study showed a high prevalence of musculoskeletal disorders, and the most affected regions were the lumbar spine, shoulder, and neck. The spinal symptoms caused the greatest DLA impairment and were the most frequent reason for seeking medical assistance, which suggests that disorders in this region were severe.

Previous history of sick leave due to MSS was the strongest variable associated with the presence of symptoms in several body regions. This result shows the importance of preventive programs designed for hospital work environments in order to control more severe musculoskeletal consequences among nursing professionals such as those identified in the present study.
